# Small (<1 cm) incidental echogenic renal cortical nodules: chemical shift MRI outperforms CT for confirmatory diagnosis of angiomyolipoma (AML)

**DOI:** 10.1007/s13244-014-0323-7

**Published:** 2014-03-08

**Authors:** Nicola Schieda, Leonard Avruch, Trevor A. Flood

**Affiliations:** 1 Department of Medical Imaging, The Ottawa Hospital, The University of Ottawa, 1053 Carling Avenue, Ottawa, Ontario Canada K1Y 4E9; 2 Department of Anatomical Pathology, The Ottawa Hospital, The University of Ottawa, 501 Smyth Road, Ottawa, Ontario Canada K1H 5L6

Non-calcified echogenic renal cortical nodules are commonly detected with abdominal ultrasound (US). The majority of these nodules represent benign angiomyolipomas (AMLs), which are present in 0.3–2.1 % of the population at autopsy [1]. The increased echogenicity (in the absence of calcification) of renal AML is due to the presence of gross or mature fat within the nodule [2]. Although renal AMLs are typically markedly echogenic, echogenicity may vary depending upon the relative proportion of fat, smooth muscle and blood vessels within the nodule [3, 4]. Renal cell carcinoma (RCC) is traditionally considered to be less echogenic than renal cortical parenchyma, although the echogenicity of RCC varies with its size. Forman et al. [5] demonstrated that one-third of RCCs less than 3 cm in size are as echogenic as “classic” AML. In a recent meta-analysis, Farrelly et al. [6] demonstrated that nearly half of small RCCs are more echogenic than renal cortical parenchyma and 11.5 % are as echogenic as renal sinus fat. The increased echogenicity of small RCC is attributed to cell arrangement with increased internal interfaces and the presence of internal degeneration or haemorrhage [7]. Nodule heterogeneity, intratumoural cysts and the presence of a hypoechoic rim are specific sonographic findings that favour echogenic RCC; while posterior acoustic shadowing is a specific sonographic finding that favours AML [6]. Although these differentiating sonographic findings are specific, they lack the sensitivity required to discriminate between AML and RCC when a small echogenic renal cortical nodule is detected in everyday practice [6].

**Fig. 1:**
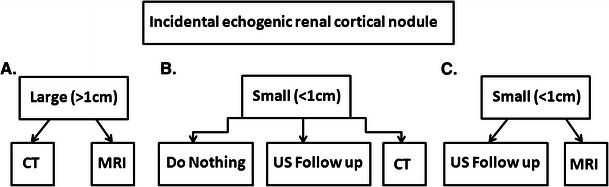
Diagnostic algorithms for incidentally detected echogenic renal cortical nodules. **a** For nodules >1 cm in size, definitive characterisation with CT or MRI to confirm the presence of gross fat and the diagnosis of AML is widely accepted. **b** For nodules <1 cm in size, management varies and is controversial. Options include: doing nothing, sonographic follow-up to confirm stability in size or CT to confirm the presence of gross fat and the diagnosis of AML. In our experience, the majority of nodules <1 cm in size cannot be further characterised with CT, which can lead to a variety of subsequent examinations including US or CT follow-up, contrast-enhanced CT or MRI. **c** In our opinion, the radiologist confronted with an incidental echogenic renal nodule measuring <1 cm in size should consider either US follow-up to confirm stability, or if definitive characterisation is required, chemical shift MRI and not CT should be performed

Given that small RCCs are commonly echogenic and may mimic renal AMLs at US and that sonographic differentiating features are insensitive, an imaging quandary occurs. Confirmatory imaging with computed tomography (CT) is generally accepted for larger lesions to detect the presence of gross fat and confirm the presumed diagnosis of AML (Fig. 1). For smaller lesions (<1 cm), recommendations vary considerably and management is controversial (Fig. 1). Some radiologists recommend no further follow-up imaging, considering them all small AMLs [2]. Sonographic follow-up is prescribed by others, since small incidentally discovered renal nodules grow slowly [8] and are associated with a low risk of metastatic disease [9]. However, metastatic disease from small RCC does occur [9] and both small RCC and AML can grow slowly [2], making differentiation based on growth difficult. A meta-analysis on the topic by Farelly et al. [6] concluded that, based on the limited available literature, all incidentally detected echogenic renal cortical nodules undergo confirmatory imaging with CT. At our institution, we observe a variety of practice patterns amongst radiologists, with many recommending confirmatory CT for echogenic renal cortical nodules measuring <1 cm in size.

**Fig. 2:**
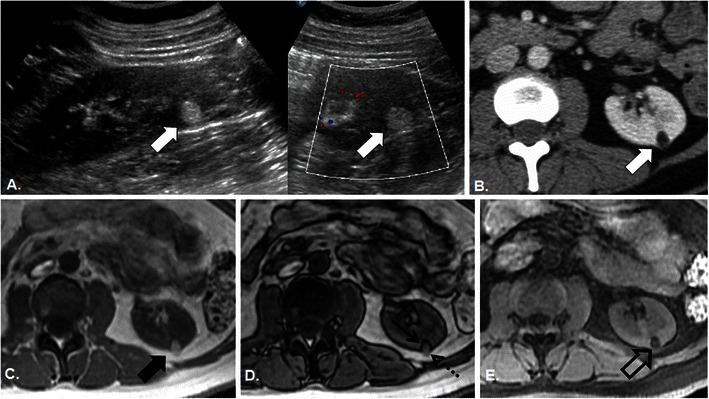
A 45-year-old woman with incidental echogenic renal cortical nodule. Sagittal and transverse grey scale (**a**) and colour (**b**) images of the left kidney depict a homogeneously hyperechoic nodule in the lower pole (*white arrows*), which measures 10 × 12 × 14 mm in size. Axial contrast-enhanced CT image (**b**) confirms the presence of a low-density nodule in the lower pole of the left kidney that measures −42 HU, diagnostic of gross fat and AML. Axial T1-weighted in-phase (IP) (**c**), opposed-phase (OP) (**d**) and spectral fat suppressed (FS) (**e**) gradient recalled echo (GRE) images demonstrate that the AML is isointense to retroperitoneal fat on IP (*solid black arrow* in **c**) and demonstrates loss of with FS (*open black arrow* in **e**) also diagnostic of gross or mature fat. Similarly, gross fat is diagnosed on the OP (**d**) by noting “india ink” or “etching” artefact at the margin of the nodule and the adjacent kidney (*black arrow*) and the absence of etching artefact at the interface of the nodule with retroperitoneal fat (*dotted arrow*)

The diagnosis of AML with CT is predicated on the ability to demonstrate gross fat within the nodule, which is essentially pathognomonic of AML; with only rare case reports of gross fat within both papillary and clear cell RCC [10]. A representative region of interest (ROI) measurement within the nodule measuring of fat density (less than −10 to −20 Hounsfield units [HU]) is considered diagnostic of AML [11, 12]. For larger nodules this is readily accomplished and an accurate diagnosis of AML is achieved (Fig. 2). For smaller nodules (<1 cm), particularly when the nodule is embedded within renal cortical parenchyma, this strategy repeatedly fails for two principle reasons: (1) inability to accurately place a representative ROI measurement due to lesion size and (2) volume averaging of imaging voxels containing an admixture of renal parenchyma and lesional fat, which increases Hounsfield unit density. This problem is worsened with larger voxel sizes; for example, when using a thicker slice reconstruction interval. If contrast-enhanced CT is performed, enhancement cannot be accurately assessed for similar reasons. For the confirmation of gross fat, CT pixel mapping can increase the diagnostic yield; with the requirement of four adjacent pixels measuring less than −10 HU improving accuracy [11]. However, in these authors’ experience, CT pixel mapping is also commonly non-diagnostic in small renal nodules due to averaging of lesional fat and renal parenchyma and is generally under-utilised by interpreting radiologists. The next step after a non-diagnostic CT varies, but could potentially result in: (1) US or CT follow-up to confirm stability and assess for growth, (2) contrast-enhanced CT (if this was not performed from the outset) to assess for possible enhancement or (3) magnetic resonance imaging (MRI) (Fig. 1). The addition of each subsequent imaging test adds undue imaging costs and unnecessary morbidity for the patient.

The MRI diagnosis of AML also relies on the ability to detect gross or macroscopic fat within the nodule. Gross fat can be detected by comparison of T1 signal intensity with and without the application of a variety of fat suppression techniques, with the suppression of signal intensity within the nodule diagnostic of gross fat [13]. Using this technique, similar diagnostic accuracy can be achieved when compared with CT for larger lesions. For the diagnosis of small (<1 cm) lesions, fat suppressed MRI techniques (like CT) may also fail to detect gross fat; with limitations related primarily to spatial mis-registration from varying breath-holds between pulse sequences.

**Fig. 3:**
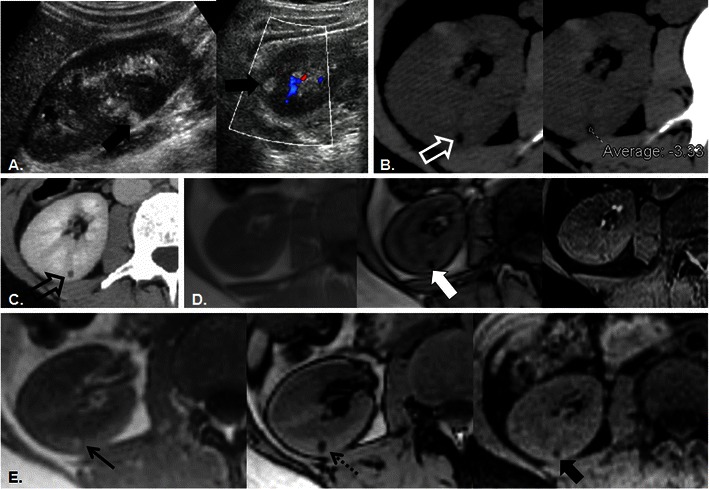
A 36-year-old man with incidental echogenic renal cortical nodule. Sagittal and transverse grey scale and colour (**a**) images of the right kidney depict a homogeneously hyperechoic nodule in the interpolar region (*white arrows*) which measures 8 × 6 × 6 mm in size. Axial unenhanced CT image (**b**) confirms the presence of a low-density nodule (*open white arrow*) with an ROI measurement of −3 HU. Pixel mapping was not performed and a follow-up CT was recommended. Six-month follow-up contrast-enhanced axial (**c**) CT image re-demonstrates the small renal cortical nodule (*open white arrow*), which is not changed in size but remains indeterminate; MRI was recommended. Axial T1-weighted IP, OP, and FS GRE images (*left to right* in **d**) reveal the renal nodule as a punctate focus of signal loss on OP image only (*white arrow*). The lesion was again considered indeterminate and follow-up was recommended. Axial T1-weighted IP, OP, and FS GRE images (*left to right* in **e**) performed 3 years later confirm the diagnosis of renal AML with persistent signal loss on the OP image (*dotted arrow*) and loss of signal intensity now noted on FS (*thick black arrow*) compared with IP (*thin black arrow*) T1-weighted images. In retrospect, the diagnosis was apparent on initial MRI examination (**d**); signal loss on OP imaging in uniformly echogenic nodules measuring <1 cm in size is confirmatory of AML

**Fig. 4:**
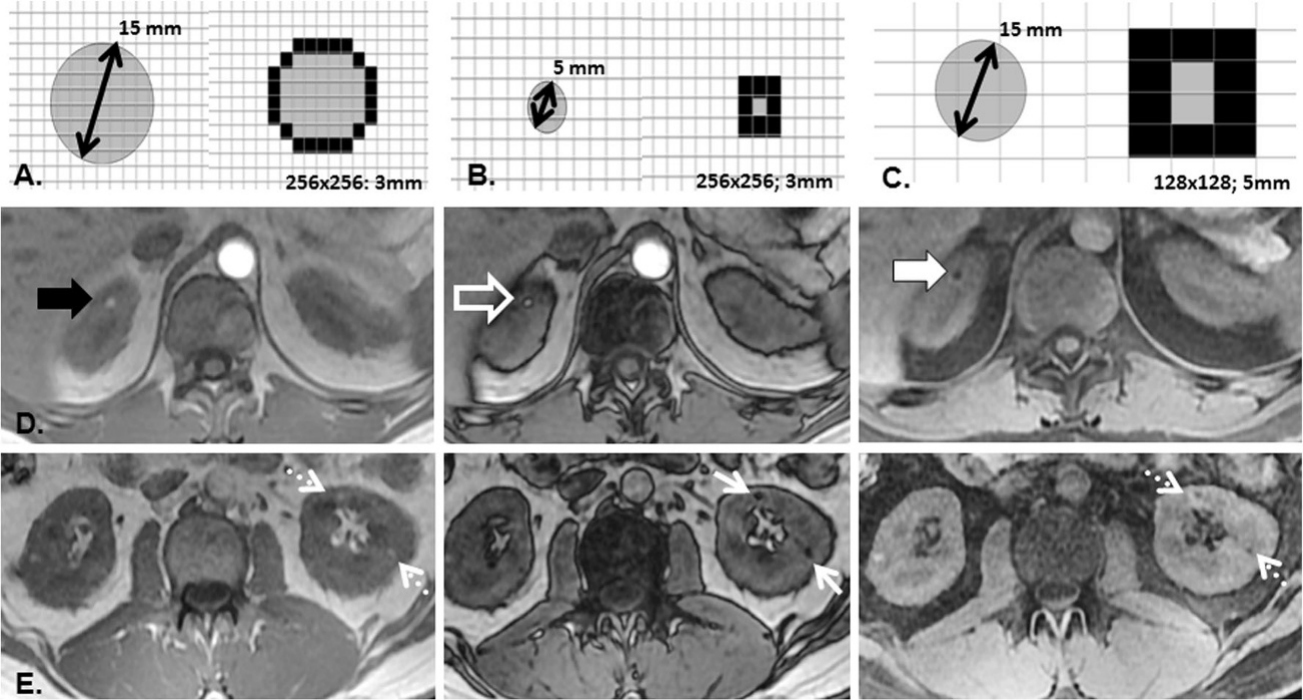
Schematic representation of renal AML diagnosis on OP MRI. In larger lesions (**a**) the presence of india ink artefact around a nodule embedded within the renal parenchyma is diagnostic of gross fat. If the nodule is extremely small (**b**), india ink artefact may obscure the entire lesion and the AML will appear only as a spot of signal loss within the renal parenchyma. Similarly, if the base voxel resolution is too large (**c**), india ink artefact may obscure the centre of the lesion so that the entire lesion appears as a signal loss. A clinical example illustrates these principles in a 38-year-old woman with tuberous sclerosis. A small (5 mm) AML is present in the upper pole of the right kidney on axial T1-weighted IP, OP, and FS GRE images (*left to right* in **d**). The AML is depicted as a focus of increased T1 signal intensity on IP (*black arrow*) with an etching artefact around its circumference on OP (*open black arrow*) imaging and which loses signal intensity with chemical fat suppression (*white arrow*). The lesion was only prospectively identified on the opposed phase image and was indeterminate at CT (not shown). In the same patient at a lower level (**e**), two tiny AML (<5 mm in size) in the lower pole of the left kidney are only prospectively identified as areas of signal loss on the opposed phase images (*white arrows*) but in retrospect also demonstrate signal intensity loss with fat suppression (*dotted arrows*). A similar phenomenon will be seen if a larger voxel size is used (*see* Fig. 3)

The use of in-phase (IP) and opposed-phase (OP) chemical shift imaging (CSI) can also be used to diagnose AML [12]. CSI exploits the different precessional frequencies of lipid and water protons by imaging with gradient recalled echo (GRE) at a pre-selected echo time (TE) when lipid/water protons are either aligned (signal intensity is additive—referred to as IP) or opposed (signal intensity cancels—referred to as OP) [14]. Imaging voxels located at the interface between water and fat tissues contain an admixture of both lipid and water protons which results in a signal loss within those imaging voxels when imaged OP. This results in a dark line surrounding a structure composed of primarily water protons (for example, the kidney) that is surrounded by primarily fat (for example, retroperitoneal fat) protons and is referred to as the “india ink” or “etching” artefact [14]. A similar phenomenon occurs when a structure composed of primarily fat protons is surrounded by or embedded within a structure composed of primarily water protons. The presence of etching or india ink artefact at the interface of a renal cortical nodule within the kidney, or lack of this artefact at the interface of this nodule with retroperitoneal fat, is diagnostic of gross fat and therefore AML [12] (Fig. 2). Within larger renal AML composed of varying amounts of fat, smooth muscle and vessels; etching artefact within the nodule is diagnostic of a gross fatty component [12]. In these authors’ experience, this imaging finding can reliably diagnose AML of all sizes including tiny AML measuring only a few millimetres (mm) in size, where CT and fat suppressed MRI frequently fails (Figs. 3, 4). Whereas the averaging of lesional fat and renal parenchyma within imaging voxels renders density measurements inaccurate, this limitation of CT forms the basis of diagnosis with chemical shift MRI. An additional benefit of chemical shift MRI compared with CT for the characterisation of echogenic renal nodules is that it is non-ionising, sparing radiation dose to this population of patients who are being worked-up for incidentally detected imaging findings. At our institution, a 10-min non-gadolinium enhanced protocol (designed for the characterisation of adrenal nodules) can be used to confirm the diagnosis of AML in <1 cm echogenic renal cortical nodules and consists of: breath-hold axial and coronal T2-weighted single-shot fast spin echo localiser sequences, breath-hold 2D and 3D IP and OP CSI, and breath-hold axial 3D T1-weighted chemical fat suppressed gradient recalled echo.

Important considerations for diagnosis of AML with CSI include: nodule size, base voxel resolution, and the relative amount of fat and water in each imaging voxel. If the nodule is extremely small (<5 mm) and/or the base voxel resolution (voxel size) is large, india ink artefact may completely obscure the centre of the nodule such that only a spot of signal loss (where the renal nodule is located) is appreciated on the OP image (Fig. 4). It is essential not to confuse signal loss from india ink artefact surrounding and obscuring a tiny lesion from signal loss within a larger renal mass. Signal loss on OP imaging from the presence of intracellular or microscopic lipid within a renal mass is not specific for AML, known to occur in clear cell RCC and recently been shown to occur in other renal masses including papillary RCC [15, 16]. It has been previously suggested that intracellular lipid content is a feature of minimal fat AML; however, this finding is not specific and, given the overlap of other imaging features (such as homogeneous low T2 signal intensity) with papillary RCC, prospective diagnosis is not possible [16–18]. The US appearance of minimal fat AML has not, to our knowledge, been described; however, it can be concluded that should a small minimal fat AML appear echogenic at US and undergo further characterisation with CT or MRI without demonstration of macroscopic fat, it should be treated as any other indeterminate solid renal mass.

With CSI, the degree of signal loss on OP imaging is dependent solely on the relative amounts of fat and water within each imaging voxel. One can therefore expect a greater loss of signal (signal void) at the interface of large (Fig. 2) or even small lesions with adequate base voxel resolution (Fig. 4) and adjacent renal parenchyma where voxels contain a relatively equal proportion of fat and water protons and signal intensity cancels. If the nodule is extremely small or with larger voxel sizes, the degree of signal loss may vary in proportion to the relative amount of fat or water protons contained within a particular imaging voxel. In our experience, even in the smallest of AML, the detection of a signal loss on OP imaging is possible and the diagnosis of AML can be established.

In conclusion, the incidental finding of an echogenic renal cortical nodule is a commonly encountered clinical scenario with an imaging differential diagnosis of renal AML or small RCC. Differentiating sonographic features are specific but lack sensitivity. Management strategies vary and are controversial. Based on the best available evidence, CT has been recommended for further characterisation of all echogenic renal cortical nodules regardless of size. While this is generally widely accepted for larger nodules, whether CT is required for the characterisation of all lesions (including those <1 cm) is uncertain due to a lack of available evidence. In the opinion of these authors, if further imaging is requested or performed for small (<1 cm) lesions, chemical shift MRI and not CT is the preferred imaging test.
